# ‘It’s not just all about the fancy words and the adults’: Recommendations for practice from a qualitative interview study with children and young people with a parent with a life-limiting illness

**DOI:** 10.1177/02692163221105564

**Published:** 2022-06-29

**Authors:** Steve Marshall, Rachel Fearnley, Katherine Bristowe, Richard Harding

**Affiliations:** Florence Nightingale Faculty of Nursing, Midwifery & Palliative Care, Cicely Saunders Institute of Palliative Care, Policy & Rehabilitation, King’s College London, London, UK

**Keywords:** Child, adolescent, family, parents, bereavement, critical illness, qualitative research, interview

## Abstract

**Background:**

Healthcare professionals report challenges in supporting dying patients who have dependent children. These parents are often uncertain how to meet the needs of their children and require appropriate support from professionals. There is limited evidence based guidance for professionals around this issue, which is informed by the views and experiences of children themselves.

**Aim:**

To develop an understanding of the perspective of children on living with parental life-limiting illness and inform recommendations for healthcare professionals.

**Design:**

Qualitative semi-structured interviews were conducted, with thematic analysis of the data.

**Setting/participants:**

A diverse sample of 32 children aged 6–17, whose parent was living with life-limiting illness, were recruited from across the United Kingdom.

**Results:**

Despite the challenges of living with a parent with a life-limiting illness, the children display agency in their response. The children: feel a responsibility to look after their family; negotiate a relationship with healthcare; employ strategies to maintain some normality; and ensure that the inevitable sadness does not become overwhelming.

**Conclusions:**

Five recommendations for healthcare professionals were developed from the findings. Clinicians should encourage dying parents to: *(1)* acknowledge the agency of children; *(2)* recognise children’s caregiving roles; *(3)* engender children’s trust in healthcare; *(4)* maintain some normality; and *(5)* discuss emotions with their children. Implementing these recommendations will assist parents with a life-limiting illness to provide evidence-based support to their dependent children.

**What is already known about the topic?**

- Healthcare professionals report challenges in supporting patients with a life-limiting illness who have young children.
- Parents with life-limiting illnesses are often uncertain of how to meet the needs of their dependent children and there is a lack of evidence on what these vulnerable children view as appropriate support.
- The death of a parent during childhood can have a long-term, negative impact, often extending into adulthood.

**What this paper adds?**

- Children have views and opinions about their parent’s life-limiting illness, which they want to be taken into account.
- Children demonstrate agency in their response to living with a parent with a life-limiting illness through: their desire to support their family; how they negotiate a relationship with healthcare; how they maintain some normality in their lives; and how they cope with the inevitable sadness and worry.

**Implications for practice, theory or policy**

- Children want the opportunity to be actively engaged when a parent has a life-limiting illness and be enabled to have a role in supporting their family.
- Recognising the role children play in the face of parental illness will encourage healthcare professionals to adopt a more holistic and inclusive approach to working with families.
- It is recommended that clinicians encourage parents with a life-limiting illness to: *(1)* acknowledge the agency of their children; *(2)* recognise the caregiving provided by their children; *(3)* try to engender their children’s trust in healthcare; *(4)* try to maintain some normality despite the illness; and *(5)* discuss emotions when communicating with their children.

## Background

In the United Kingdom (UK), parents with dependent children (under the age of 18) account for approximately 5% of all deaths.^1–3^ This figure is higher in low and middle-income countries, which account for 70% of all deaths from non-communicable diseases, and the majority of people living with HIV.^4,5^ Nationwide population-based studies have shown that the death of a parent during childhood can have a long-term effect upon psychological wellbeing,^6,7^ as well as causing lasting socio-economic disadvantage.^8^ Adults bereaved in childhood are at increased risk of poor outcomes, including anxiety, depression and anti-social behaviours.^3^

For parents with a life-limiting illness, their dependent children can be a major source of distress and anxiety,^9^ and their children’s welfare is often their paramount concern.^10^ Whilst dealing with their own emotions, these patients must also consider the impact that their disease will have on their children and are often unsure of how best to support them.^11,12^ Advice from family and friends can be conflicting, may not be objective and may not be in the best interests of the child.^13^ When parents with a life-limiting illness turn to healthcare professionals for support, guidance is not always provided.^14,15^ A consequence is that parents with life-limiting illnesses are often uncertain of how to meet the needs of their dependent children and these vulnerable children do not receive appropriate support at a highly sensitive time.^16,17^

Healthcare professionals are uniquely positioned to advise patients with a life-limiting illness about their children.^18^ However, a lack of confidence, professional detachment and concerns about causing harm can inhibit the provision of appropriate support to dying parents around their children.^19–21^ Respect for patient autonomy and building trust in the professional-patient relationship can also inhibit conversations about dependent children.^22,23^ Working with this cohort of patients can be emotive and challenging for professionals, who may avoid the topic of dependent children.^15,24^ Enquiring about children is not routine in most clinical encounters, putting the onus on parents to raise the issue.^25^ Therefore, even when a patient is dying, their dependent children can be peripheral to the care and support provided.^26,27^

The support provided to children prior to the death of a parent may be insufficient and children report feeling invisible and overlooked when their parent is ill and dying.^28^ Improving the support provided at this sensitive time may help to mitigate some of the poor long-term outcomes. Norway, Finland and Sweden are currently the only countries to have recognised the needs of children when a parent is ill or dying, and enacted legislation placing a duty on healthcare professionals to consider the needs of dependent children.^22,29–31^ They recognise their commitment to the United Nations (UN) Convention on the Rights of Children and acknowledge that children have a right to be involved in such a serious matter affecting them.^32^

An evidence review found few primary research studies with self-report data from children of a parent with a life-limiting illness ^33^; included studies were generally quite dated with a focus upon parental cancer. Although some guidance has involved children in the development through patient and public involvement (PPI), to date children have not been included as research participants to inform the content of such guidance.^23,34,35^ The aim of this research was therefore to develop an understanding of the perspective of children on living with parental life-limiting illness. The objectives were to determine the needs and preferences for pre-bereavement support of children and young people who have a parent or primary caregiver with a life-limiting illness, and to develop recommendations for practice.

## Methods

### Design

Qualitative methodology was used, with reporting following the COREQ guidelines.^36^ The research design is based upon the epistemological assumptions that children have a unique and subjective perspective and that qualitative research can gain an understanding of their perspective.^37^ Semi-structured interviews and thematic analysis were selected as appropriate methods for research questions aiming to understand how a group of people perceive and conceptualise a particular phenomenon.^38–42^

### Sampling

Inclusion criteria were children aged 6–17 who had current experience of parental life-limiting illness. Only children aged 6 and older were included, to ensure the permanence of death was understood by participants.^43^ The definition of life-limiting illness included any non-curative condition and from which a parent would be expected to die prematurely.^44^ A purposive sampling strategy was employed, strategically selecting children aged 6–17 who were deemed emotionally able by their parents and the responsible clinician to engage in an interview and discuss their experience of having a parent with a life-limiting illness. Potential participants were identified by clinical teams in 3 acute hospitals and 10 hospices across the UK. Parents of potential participants were then approached by a member of the clinical team with study details. Through discussion with the parents, clinical teams screened for additional vulnerabilities such as mental health issues or severe developmental delays. Parents who gave verbal consent for their child to participate (see ethical procedures below) were then contacted by SM, who arranged an interview time. The study was also promoted on social media and by voluntary organisations. Any parents who self-selected were contacted, screened and consented by SM.

### Data collection

All of the interviews were conducted by SM, a White British male social worker, with a relevant PhD that utilised qualitative methods. None of the participants were previously known to the research team. Participants and their parents were offered a choice of venue for the interview. An interview guide was developed from a review of the literature^33^ and from professional experience within the research team, which was explained to participants before each interview (available as a Supplemental Material). The interview schedule was pilot tested with a 10-year-old living with a parent with a life-limiting illness and then refined further. Key issues were raised in all the interviews, but were adapted to the age of the child. A distress protocol and a safeguarding plan were developed. Parents were provided with information about additional sources of support for their children and were also offered a follow-up telephone call to enquire about their children’s welfare. A reflective research diary was completed after each interview. Interviews were audio-recorded, transcribed verbatim and pseudonymised for reporting. Interview transcripts were not returned to participants.

### Data analysis

Analysis was informed by the approach to thematic analysis developed by Braun and Clarke.^40–42^ This approach is reflexive, assuming that meaning is contextual and situated, acknowledging that there are multiple realities and welcoming the active role played by the researcher in the production of knowledge. SM and RF took the lead on analysis, becoming immersed in the data, ascribing codes to ‘chunks’ of data and organising the codes into candidate themes. *Microsoft Excel* was used to manage the data. The candidate themes were refined and developed into the final themes through a process of discussion, revision and agreement with KB and RH.

### Research ethics and informed consent

Ethical approval for the study was granted in March 2019 by the NHS Health Research Authority (Bloomsbury Research Ethics Committee – Reference: 19/LO/0234). Prior to the interview, the study was explained verbally to parents and participants, parents were provided with an information sheet and age-appropriate information sheets were provided to the children. The extent of children’s understanding of the life-limiting condition was clarified with parents and they were assured that the interviewer would not give any additional information. Parents of participants aged 6–15 gave written informed consent and these participants also provided written assent. Participants aged 16 and 17 gave written informed consent. Anonymity of participants was protected by the use of pseudonyms for all names of people, places and institutions. Participants self-selected a pseudonym. Due to the sensitivity of the topic, the children were assured that they could decline to answer any question and halt the interview at any time (as per the distress protocol). At the end of the interview, the interviewer asked participants about their wellbeing and clarified how they could obtain support.

### Patient and public involvement^45^

A Palliative Care PPI Group and a Young Persons’ Research Advisory Group (YPAG) provided feedback on the study prior to funding, to gain the perspective of children and those bereaved in childhood from the onset. The YPAG subsequently helped with the development of age-appropriate information sheets for children. The research was guided by a project advisory group, which included three experts by experience (two adults who were bereaved as children and the wife of a patient living with a life-limiting illness, who has a 10-year-old child). The findings were shared with an after-school ‘Research Methods Club’, in order to obtain young people’s perspective on the meaning within the data.^46^ The YPAG also provided feedback on the five recommendations.

## Results

### Participant profile

Interviews with 32 children (see Table 1) were conducted between May 2019 and March 2020 and ranged from 18 to 50 min (median 29 min). Participants were recruited by hospitals (*n* = 18), hospices (*n* = 12), social media (*n* = 1) and a voluntary organisation (*n* = 1). Participants were interviewed on their own (*n* = 23) or with one or more parent present (*n* = 9). Interviews occurred in the participant’s home (*n* = 20) or in a private room in the hospital or hospice (*n* = 12). An additional 16 potential participants were identified or self-selected, but did not participate because: contact was lost (*n* = 8); the child decided against participation (*n* = 3); a parent decided against participation (*n* = 2); the child did not meet the eligibility criteria (*n* = 2); or the parent died prior to the interview (*n* = 1).

**Table 1.** Demographic profile of participants.

		*n* (%)
Gender	Male	16 (50)
	Female	16 (50)
Age	6–8	11 (34)
	9–11	6 (19)
	12–15	11 (34)
	16–17	4 (13)
Ethnicity	Asian or Asian British	1 (3)
	Black, Black British, Caribbean or African	5 (16)
	Mixed or multiple ethnic groups	4 (12.5)
	Other ethnic group	4 (12.5)
	White	18 (56)
Parental illness	Cancer	11 (34)
	Cystic fibrosis	9 (28)
	Renal failure	4 (13)
	MND	3 (9)
	Other	5 (16)

### Main findings

Despite the challenges associated with parental life-limiting illness, the findings reveal that children have views and opinions about their parent’s illness which they want to be taken into account, and they find ways to play an active role in the care of their parent and family. Participants in this study were not passive but displayed independent thought in how they interacted with their parent’s illness and healthcare, and made choices to influence events that had an impact upon their social world. This ‘agency’ is exhibited in four main themes drawn from the findings: looking after the family; negotiating healthcare; trying to maintain some normality; and managing the sadness. The themes are evident across the interviews, irrespective of the age or background of the participant, however nuances within the themes by age have been drawn out in the description.

### Theme 1: Looking after the family

Participants talked in detail about the impact of a parental life-limiting illness on their own life. For many, the illness resulted in a need to undertake some practical and emotional support, beyond that that might usually be expected of a young child. This was seen as an obligation by some participants, and a responsibility that required knowledge and skills to manage:

*‘cos it’s like a duty, cos you’ve gotta look after them. . .’cos you’ve gotta make sure that they’re okay. ‘cos if they’ve just come out of hospital, you get wary when you’re left alone with them because. . .especially if you have never been alone with them before, you don’t know what to do. You don’t know. . .for example. . .erm if you’re very young, you don’t know your address so you. . .so you can’t call the emergency services’*

(Timmy, aged 9)

Among the practical tasks described by older children were caregiving duties, such as helping to look after younger siblings, and undertaking household chores which impacted upon their ability to go out of the house. Although participants described many household and caregiving duties, they rarely saw themselves as a carer:

*‘I have to do much more . . . erm . . . I don’t really go out as much . . . look after my little brothers sometimes . . . do more cleaning . . . that would be me [a carer] but I won’t class myself as one’*

(Morgan, aged 14)

This sense of responsibility to look after the family extended to ensuring their own actions impacted positively upon the family. Examples included being well behaved, working hard at school and keeping happy, in order not to add any additional stress to the family at a challenging time. Participants described strategies to manage the additional responsibilities of looking after the family, in particular ensuring that they had respite from the situation at home. When at home, older participants talked about their sense of responsibility and the needs of others dominating over their desire to do things for themselves:

*‘Have as much time with your friends as you can, just doing things that make you happy, make you laugh. ‘cos that’s what you need, because when you come home and reality hits. . .it’s good to do stuff out of the house, ‘cos when you’re in the house that’s when you’re. . .there you feel like you’ve got everything to do. When you’re out of the house enjoy. . .even if it’s just going to school, it’s better than nothing’*

(Katie, aged 17)

Whilst the participants enjoyed and valued having respite, it was not always easy to achieve and often required them to stay near to home in case they were needed:

*‘if I’d go too far, I wouldn’t hear him call if he needed something’*

(Bob, aged 13)

### Theme 2: Negotiating healthcare

When a parent has a life-limiting illness, healthcare invariably becomes a part of the world of their children. This may be remote and distanced, but nevertheless healthcare has an impact on all the members of the family, including the children. As such, participants stated that they would like healthcare professionals to be more proactive and provide information about their parent’s care/treatment in language understandable by children:

*‘speak with family and children because obviously we’re children and they’re like ‘oh yeah you might not understand as much’ but take that time to sit down and tell them and explain what’s happening, obviously in like child words but just take the time to acknowledge that there’s kids in the family as well and it’s not just all about the fancy words and the adults’*

(Josh, aged 16)

Older participants also discussed the importance of the right level of knowledge about their parent’s condition for them, and the importance of being able exert some control over what they knew:

*‘I think I know enough to be comfortable, I think. . .if I knew. . .if I knew too much I’d worry a bit too much and then that would put me in like a really like worried state everyday’*

(Harry, aged 14)

However, the importance of negotiating the right level of information was underscored by experiences of younger participants too, some of whom had assumed that their parent’s condition was curative until they were told otherwise:

*‘I thought he had just got some kind of chronic illness that . . . it was serious but it like. . .it can be treated. . .like it wasn’t fatal. . .he’s just taking these drugs because he has to and it’ll eventually work’*

(Sammy, aged 10)

Such misunderstandings or inadequate information about their parent’s condition resulted in a lack of confidence in the healthcare team, as they felt that the team had failed to make their parent better. However the youngest children also demonstrated a misdirected sense of responsibility on themselves, feeling that not being there with their parent somehow negatively influenced the outcome too:

*‘sometimes when I’m not there it makes me sad. I feel like they won’t help him . . . or make him better’*

(Superman, aged 7)

Being able to have confidence in the healthcare team to look after their parent when they are not with them was particularly important. Trust and confidence were engendered through opportunities to understand the nature of care and support that the parent would receive in hospital:

*‘I don’t really feel down now ‘cos I know that my mum has a lot of support at the hospital. . .when I went with her to do her dialysis one time, I saw like doctors and er nurses. . .they like had a really good relationship. . .and like. . .more like a nice friendship basis relationship and so I knew that like they could support her with. . ..for like emotionally and like also medically as well’*

(Eagle, aged 14)

### Theme 3: Trying to maintain some normality

Having a parent with a life-limiting illness inevitably brought changes to the lives of the children and their families. Participants, particularly those in their teens, described the impact of the parental illness and how their lives were different from those of their peers. While other children looked forward to activities and holidays, children with a parent with a life-limiting illness did not have as much to look forward to:

*‘when I’m at school and like. . .people are talking about stuff that they do on the holidays with their mum. . .erm. . .and I can’t do that’*

(Morgan, aged 14)

These differences increased participants’ desire not to be seen as different from their friends and to find ways to maintain some normality. Participants did not always want their friends and school to be aware of their parent’s life-limiting illness, so would avoid conversations about their parent’s illness or their home life:

*‘I don’t really mention it at school. I mean a lot of my mates know about my dad. . . . . .y’know sometimes we get on the topic about. . .you know. . .family and stuff and it’s just. . .but like if I can avoid speaking about it in school I rather would. . .It’s just. . .you know. . .I don’t really like. . .I don’t see the point in my friends needing to know what’s going on in my personal life’*

(Harry, aged 14)

Influences of their home situation also impacted upon participants’ ability to invite friends to their home. For example, they talked about having to warn friends about their parent’s appearance and behaviour, particularly related to sickness and bodily functions:

*‘some of my friends stay over so I. . .I just kind of have to like warn them about. . .yeah dad has dementia and he has something with his stomach as well which can sometimes cause him to throw up every like night or just randomly feel sick and he throws up so I just kind of have to like, warn them about that’*

(Connie, aged 14)

Participating in activities typical of their age group was important and also provided respite from the parent’s illness, despite the additional challenges. However the parent’s illness can also create a ‘new normal’ and perceived positive benefits, such as a sense of maturity and a changed perspective on life. One participant described not having concerns about moving away from home because of the additional stress and responsibility she had experienced in her childhood:

*‘I think what’s happened has kind of prepared me a bit more for the real world. Like I am not worried about moving out next year to go to Uni, ‘cos I know I’ve managed to get through this, I can do that too’*

(Katie, aged 17)

### Theme 4: Managing the sadness

Having a parent with a life-limiting illness inevitably had a profound emotional impact upon the participants, irrespective of age. They described being defenceless against the impact and shock of events:

*‘it’s like a wave. . .it’s like some kind of wave where it just keeps building and keeps building. You know something’s gonna happen and then it suddenly does and it’s like, it washes over you. . .I don’t think about it and then something finally hits and I get shocked by it. . .and it’s not nice but there’s not much that you can do against it’*

(Sammy, aged 10)

This sense of turbulence was described even by the youngest participants. Although happy at times, the sadness and worry would return:

‘*It’s just sometimes I feel worried and other times I feel happy’*

(Mermaid’s Alive, aged 6)

However, the extent of this sadness is not always shared with others, as children often kept their sadness private due to fear of the emotional impact their sadness would have upon others:

[Referring to her mother’s illness]

Elsa: *It’s sad. . .*

SM: *What do you do when you’re sad? Do you tell anybody?*

Elsa: *No. . .I just keep it a secret. . .*

SM: *And why do you keep it a secret?*

Elsa: *‘cos I don’t want no one to know*

SM: *Oh and why not? What would happen if people found out you were*

*sad?*

Elsa: *They would cry*

SM: *And would that be bad?*

Elsa: *Yeah*

(Elsa aged 6)

Participants exhibited agency by describing the strategies they employed to manage the sadness and worry, for example the importance ascribed to having respite from the situation at home:

*‘Make sure you look after yourself. The only way you’re gonna be able to help them is if you’re able to help yourself first. Because if you’re spending all your time trying to help them, then all that’s gonna happen is you’re gonna deteriorate yourself. You’re not gonna be any help that way. . .Take a bit of time just to do something that you enjoy. Make sure that you’re not spending all your time worrying’*

(Katie, aged 17)

## Discussion

The findings of the study reveal that children have views and opinions about their parent’s illness, and want to have control over the extent of information they have. Children want age appropriate information to help them to adjust to the presence of the illness in their lives, and want parents and professionals to help them to achieve this by engaging with them directly and through resources. The emotional and social impact for children with a parent with a life-limiting illness is significant, and children describe the importance of normal activities in managing that impact. Respite from the home situation helps them to maintain normality, and increase their resolve to manage their home life and the additional responsibilities they take on. Being actively engaged with, and retaining a degree of control over their actions and responses to the illness, helps children to maintain agency in the face of parental life-limiting illness

Not only are these findings consistent with other research in the field,^33,47,48^ but accord with the UN Convention on the Rights of the Child,^32^ childcare legislation in the UK^49,50^ and prevailing theories of childhood,^51^ which all advocate for the meaningful contribution of children to their own lives. Five recommendations for healthcare professionals have been generated from the findings, which are summarised in Table 2 and are discussed below.

**Table 2.** Five recommendations for healthcare professionals, when working with patients with a life-limiting illness who have dependent children.

*Agency*	Acknowledge children’s desire to make an active contribution when a parent has a life-limiting illness
*Caring*	Recognise children’s caregiving role and their desire to look after the family when a parent is unwell
*Trust*	Explain children’s need to have confidence in the professionals caring for their parent
*Normality*	Reinforce children’s need to maintain some normality and have respite from the parent’s illness
*Emotions*	Support parents to have a discussion with their children around how they feel about their parent’s illness

### Agency

Participants in this study show agency in their response to parental illness. However acknowledging the role that children play when a parent has a life-limiting illness may be challenging for professionals and can require reflection on their values around the rights of children, particularly when they are very young.^18,33,52^ In their interactions with parents with a life-limiting illness, healthcare professionals are encouraged to initiate a discussion around children’s desire to be actively involved and the roles they could or do play within the family depending on their age. Portraying children as active participants may encourage parents to adopt an approach that their children will find supportive and appropriate and which acknowledges the contribution they make to the family.

### Caring

The findings have revealed that children of all ages feel a sense of responsibility towards their parent with a life-limiting illness and want to look after the whole family. Children can be invisible carers, with their contribution to their parent’s care often unseen and unrecognised.^53,54^ Professionals are advised to highlight children’s contribution to parents and make suggestions around how children can fulfil their sense of responsibility. Whilst balancing their need to maintain some normality, offering children opportunities to be involved in providing support may have a beneficial impact.

### Trust

Although the children of patients with life-limiting illnesses can be distanced from the healthcare setting, these findings have shown the impact of healthcare professionals and institutions on their world. Children need to be able to trust and have confidence in the professionals caring for their parent.^22^ Helping children to learn more about the healthcare their parent is receiving is one way to engender trust. Directing parents to useful age appropriate resources for their children about hospitals and healthcare,^55–57^ or encouraging open and honest conversations about care and treatment, may help children feel confident in the care their parent is receiving.

### Normality

Other studies have shown children’s need to maintain some normality and have respite from their parent’s illness.^54,58^ This study has substantiated these findings. In their interactions with parents with a life-limiting illness, professionals can make reassurances that having time away from home and engaging in everyday childhood activities is appropriate and beneficial. Continuing to attend school and maintain a routine is to be encouraged.

### Emotions

Emotions were discussed by all participants, and the terms ‘sadness’ and ‘worry’ permeated the interviews. Parents should be encouraged to have open discussions with their children about their emotional response to their parent’s illness, using words such as sadness and worry as appropriate, to help children to know that all emotional responses are normal. Acknowledging that not all children will want to talk to their parents is also important. Children should be reassured that it is ok to talk about their emotions to other people too. Directing parents towards appropriate resources that specifically address emotions may be helpful.^59,60^

## What this study adds

A recent review revealed that there are few studies asking children to self-report their experience of living with parental life-limiting illness.^33^ This study has enabled a diverse sample of children to articulate their perspective when a parent is dying. Based upon this primary data, the first evidence-based recommendations have been developed to support healthcare professionals working with patients with a life-limiting illness who have dependent children.

## Further research

Data in this study was collected prior to the Covid-19 pandemic. The pandemic has had an impact on children’s involvement when a parent is dying,^16,61^ and this warrants further investigation. The pandemic has necessitated the use of virtual communication with family members^62^ and research into how children can be engaged virtually may be fruitful. In addition, more depth of understanding could be gained by exploring experiences by stage of illness. Longitudinal work with children of parents with a life limiting illness would add further depth of understanding about how children’s experiences, views and preferences change over time and as their parent’s illness progresses.

## Strengths and limitations of the study

Two recent reviews identified a lack of diversity in studies of children facing the death of a parent.^15,19^ This study has the largest reported sample of a qualitative study involving children affected by parental life-limiting illness. Over half of the sample are pre-teens, 44% are from Black, Asian and ethnically diverse backgrounds and a variety of parental illnesses are represented. However recruiting through clinical teams and requiring parental consent may have resulted in gatekeeping by adults.^63^ As children are viewed as potentially vulnerable, our ethical approval for the study required an assessment of their emotional state prior to them being approached about the study. As such, this may bias our findings and limit their transferability to those deemed to be ‘emotionally able’ by an adult. Despite a robust approach to PPI, the study would have been enhanced by including a child as co-applicant, ensuring the perspective of children was intrinsic to the research.

## Conclusion

This study has produced evidence-based guidance for all professionals working with patients with a life-limiting illness who have dependent children (summarised in Table 2). By ensuring that the five recommendations are incorporated into their practice, clinicians can provide support to dying parents that that is inclusive and beneficial to their dependent children.

## Supplemental Material

sj-pdf-1-pmj-10.1177_02692163221105564 – Supplemental material for ‘It’s not just all about the fancy words and the adults’: Recommendations for practice from a qualitative interview study with children and young people with a parent with a life-limiting illness Click here for additional data file. Supplemental material, sj-pdf-1-pmj-10.1177_02692163221105564 for ‘It’s not just all about the fancy words and the adults’: Recommendations for practice from a qualitative interview study with children and young people with a parent with a life-limiting illness by Steve Marshall, Rachel Fearnley, Katherine Bristowe and Richard Harding in Palliative Medicine
