# Supplementary material for: ‘It’s not just all about the fancy words and the adults’: Recommendations for practice from a qualitative interview study with children and young people with a parent with a life-limiting illness
Source: Palliat Med. 2022 Jun 29;36(8):1263–72. doi: 10.1177/02692163221105564 (PMC9446426; doi:10.1177/02692163221105564)
Supplement: sj-pdf-1-pmj-10.1177_02692163221105564 – Supplemental material for ‘It’s not just all about the fancy words and the adults’: Recommendations for practice from a qualitative interview study with children and young people with a parent with a life-limiting illness [file sj-pdf-1-pmj-10.1177_02692163221105564.pdf]

Interview Guide

1. Introduction	2. Tell me about yourself	3. Parental Illness	4. Being told
• Assent form<br>• Safeguarding<br>• Can stop<br>• Upsetting	• Age<br>• School<br>• Interests	• Condition<br>• Understanding<br>• Impact on parent<br>• Treatment	• How did you find out?<br>• Who told you?<br>• How did you feel?<br>• Questions?
5. Information	6. Professionals	7. Changes	8. Caring role
• Tell anybody?<br>• Did you try to find out about the illness?<br>• Is there anyone else you would like to talk to?	• Met any?<br>• What do they do?<br>• Ask them questions?<br>• Pall care or hospice team	• Any changes?<br>• Changes in parent<br>• Changes in activities<br>• Changes in routine	• Tasks<br>• Time spent<br>• Feelings
9. School	10. Feelings	11. Positives	12. Wrap up
• Impact or changes?<br>• Friends<br>• Teachers	• How do you feel about the illness?		• How are you feeling now?<br>• Anything extra?<br>• Any worries?<br>• Choose a pseudonym<br>• What happens now<br>• How to make contact
